# Molecular docking analysis of 2009-H1N1 and 2004-H5N1 influenza virus HLA-B*4405-restricted HA epitope candidates: implications for TCR cross-recognition and vaccine development

**DOI:** 10.1186/1471-2105-14-S2-S21

**Published:** 2013-01-21

**Authors:** Chinh TT Su, Christian Schönbach, Chee-Keong Kwoh

**Affiliations:** 1 Bioinformatics Research Centre, School of Computer Engineering, Nanyang Technological University, Singapore 639798; 2 Biomedical Engineering Research Centre, College of Engineering, Nanyang Technological University, Singapore 637553; 3 Department of Bioscience and Bioinformatics, School of Computer Science and Systems Engineering, Kyushu Institute of Technology, Fukuoka 820-8502, Japan; 4 Biomedical Informatics Research Center, Kyushu Institute of Technology, Fukuoka 820-8502, Japan

## Abstract

**Background:**

The pandemic 2009-H1N1 influenza virus circulated in the human population and caused thousands deaths worldwide. Studies on pandemic influenza vaccines have shown that T cell recognition to conserved epitopes and cross-reactive T cell responses are important when new strains emerge, especially in the absence of antibody cross-reactivity. In this work, using HLA-B*4405 and DM1-TCR structure model, we systematically generated high confidence conserved 2009-H1N1 T cell epitope candidates and investigated their potential cross-reactivity against H5N1 avian flu virus.

**Results:**

Molecular docking analysis of differential DM1-TCR recognition of the 2009-H1N1 epitope candidates yielded a mosaic epitope (KEKMNTEFW) and potential H5N1 HA cross-reactive epitopes that could be applied as multivalent peptide towards influenza A vaccine development. Structural models of TCR cross-recognition between 2009-H1N1 and 2004-H5N1 revealed steric and topological effects of TCR contact residue mutations on TCR binding affinity.

**Conclusions:**

The results are novel with regard to HA epitopes and useful for developing possible vaccination strategies against the rapidly changing influenza viruses. Yet, the challenge of identifying epitope candidates that result in heterologous T cell immunity under natural influenza infection conditions can only be overcome if more structural data on the TCR repertoire become available.

## Background

In 2009, the outbreak of a new swine-origin strain of influenza A H1N1 caused widespread human infection [1]. One of the most important surface proteins, hemagglutinin (HA) permits the virus to bind to cell membrane and infect the cells. Since mutations enable the virus to escape from either T cell or antibody recognition, current flu vaccines were not effective against the emerging virus. Sequence analyses showed that the HA sequence of the pandemic 2009-H1N1 underwent an antigenic shift [2] that altered its antigenicity in context of the seasonal flu vaccine.

The antigenicity of HA 2009-H1N1 remained highly conserved to pandemic 1918-H1N1 and partially conserved to seasonal flu strains of the 1930s. Therefore, the majority of infected individuals who were vaccinated with the WHO recommended seasonal flu vaccine did not produce neutralizing antibodies against the new influenza strain. However, elderly and individuals born before 1950 were less affected than expected. The lower infection rate of these age groups has been interpreted as the results of cross-reactive T cells [3] and antibody [4] responses to the pandemic 1918-H1N1 and partially cross-reactive T cell response to seasonal flu strains of the 1930s. A study by Boon *et al. *[5] on CD8^+ ^T cell recognition of heterosubtypic H1N1 variants indicated that repeated infection with heterologous viruses may increase cross-reactive Cytotoxic T Lymphocytes (CTL) and thus confer protection against newly emerging strains in absence of a cross-reactive antibody response. Further support for this concept comes from a study of subjects who were vaccinated against seasonal influenza and showed *in vitro *cross-reactive T cell responses against HA of the pandemic 2009-H1N1 [6].

In growing recognition of the role of T cell responses to H1N1, several groups conducted large-scale Human leukocyte antigen (HLA) binding motif scanning analyses of pandemic and seasonal strains to predict and identify conserved peptides that elicit cross-reactive HLA class I and/or class II restricted T cell responses [7-9]. While the affinity-based approach allows a broad coverage of HLA supertypes and epitope bindings [7,8], structural approach gives better insight view onto T cell recognition of the HLA-restricted T cell epitopes [10-12]. In our study, we are interested in immunogenicity that depends on the quality of T-cell receptor (TCR) interaction with the HLA/peptide complexes rather than HLA-binding peptide affinity only. We, therefore, combined affinity-based epitope prediction with molecular docking to generate conserved high confidence HA T cell epitope candidates of current and past pandemic strains, and consequently analyzed the potential TCR cross-recognition of 2009-H1N1 and 2004-H5N1.

According to Archbold *et al. *complex of DM1-TCR and HLA-B*4405/peptide showed significant enhancement in T cell-mediated responses among micropolymorphisms in the HLA-B*44 family, and as such they are key factors in controlling persistent viral infections [12]. Thus, to perform the experiments we used HLA-B*4405 and DM1-TCR as models. Results of structural models of TCR cross-recognition between 2009-H1N1 and 2004-H5N1 revealed steric and topological effects of TCR contact residue mutations on TCR binding affinity. While these results are novel with respect to HLA-B*4405-restricted H1N1 HA epitopes and DM1-TCR, yet with limited available structural data upon the TCR repertoire, more investigations and experimental analyses are still recommended for further broad perspective of their utility in vaccine development against the emerging virus strains.

## Results and discussion

### Conserved HA T cell epitope candidates generated for HLA-B*4405 and DM1-TCR

Examining various HA sequences among the pandemic 2009-H1N1, 1918-H1N1, and WHO recommended vaccine strains [Additional file 1], we predicted potential 9-mer T cell epitopes using the tool NetCTL v1.2 from Immune Epitope Database (IEDB) (see Methods). Nineteen candidates, which scored greater than 0.75, are more likely well presented by HLA-B44 family [Additional file 2]. We then docked these 19 epitopes to HLA-B*4405, and subsequently the HLA-B*4405/epitope complex as ligand to DM1-TCR. The 19 predicted epitope candidates differ in their physico-chemical properties. Eight candidates starting at positions 128, 188, 251, 131, 229, 400, 240, and 259 contain mostly hydrophobic and polar residues, whereas other 11 candidates (positions 50, 412, 446, 463, 493, 514, 421, 475, 482, 81, and 274) are composed of predominantly hydrophilic residues [Additional file 3] which result in higher HLA-B*4405 binding energies (Table 1). As expected, all the 19 epitopes bound to the helical antigen-presenting groove of the HLA-B*4405 [13]. Eleven epitopes (50, 128, 251, 412, 463, 493, 514, 131, 229, 400, and 259) bound in similar orientations to HLA-B*4405 with Glu at position 2 and residues at the C-terminus binding to the B and F pockets of the HLA-B*4405, respectively [13-15]. Residues which occupy the centre of the binding groove are solvent-exposed and likely to bind to TCR [13,16]. In contrast, most residues of epitopes at positions 188, 446, 421, 475, 482, and 240 interacted with the HLA-B*4405 peptide binding pockets. In our model, the deeply embedded residues were not exposed to the solvent. Therefore TCR recognition of HLA-B*4405/epitope complexes is expected to be conformationally restrained.

**Table 1:** Total binding energies of the 19 epitope candidates (in descending HLA-B*4405 binding energy ranks).

Position	Sequence	Docked to HLA-B*4405 Docked to DM1-TCR			
		
		Energy	Rank	Energy	Rank
240	QEGRMNYYW	-1,006	1	-541.7	17
188	KEVLVLWGI	-921.8	2	-573.9	13
128	FERFEIFPK	-869.9	3	-594.6	12
482	FEFYHKCDN	-846.8	4	-743.9	2
259	FEATGNLVV	-815.2	5	-608.9	10
400	IEKMNTQFT	-775.8	6	-688.5	5
131	FEIFPKTSS	-752.9	7	-560.5	14
251	VEPGDKITF	-743.3	8	-599.1	11
229	PEIAIRPKV	-687.7	9	-548.9	15
50	LEDKHNGKL	-661.4	10	-552.1	16
274	MERNAGSGI	-644	11	-	-
514	REEIDGVKL	-643.8	12	-666.3	7
81	PECESLSTA	-633.3	13	-	-
446	LENERTLDY	-603.1	14	-621.1	9
412	KEFNHLEKR	-599.5	15	-737.2	3
493	MESVKNGTY	-543	16	-645	8
475	KEIGNGCFE	-519.6	17	-715.4	4
463	YEKVRSQLK	-475.3	18	-751.3	1
421	IENLNKKVD	-464.6	19	-666.7	6

DM1-TCR was docked to the HLA-B*4405/epitope complexes to assess their binding conformation. In the models with minimum binding energies, DM1-TCR V_α _and V_β _domains interacted with HLA-B*4405 α_1 _and α_2 _domain residues and solvent exposed residues of the epitopes. Epitope candidates 240 and 482 were embedded in the binding groove of HLA-B*4405, and interacted with DM1-TCR via Tyr^7 ^and Tyr^8 ^(epitope 240) and Lys^6 ^(epitope 482) as shown in Table 2. These interactions appeared to be mediated by a change of the TCR binding position to HLA-B*4405 α_1_- and α_2_-domain residues, resulting in contacts with polar and charged residues Asn^70^, Glu^76^, and Gln^155 ^of the HLA-B*4405. Hydrogen bond lengths (in Å) and dihedrals around these H-bonds made by DM1-TCR and HLA-B*4405 domains in cases of exposed and embedded epitope types were calculated. In case of epitope 259, residues at positions 4, 5, and 6 were exposed while the TCR V_α _domain interacted with Asn^70 ^(2.1 Å) and Glu^76 ^(2.2 Å). In contrast, for the embedded-type epitope 240, we observed binding of TCR V_β _domain to Asn^70 ^and Glu^76 ^at 2.5 Å H-bond lengths. Epitope candidates 400 (exposed-type) and 482 (embedded-type) also showed similar noticeable H-bond length changes. Distances of the TCR to the residues Gln^155 ^(HLA-α_2_) decreased when the contacts with the HLA domains shifted from the TCR V_β _to TCR V_α_. Meanwhile, the dihedrals between residues that made the H-bonds also showed decreasing trend when the TCR changed its binding positions to the HLA-B*4405 from V_β _to V_α _domain (e.g. from -133.7^° ^to -54.1^° ^in cases of epitope 240 and 259 respectively) [Additional file 4]. It could suggest that the changes in H-bond lengths and dihedral angles indicate an attempt of the TCR to adjust its binding access to the HLA-B*4405/epitope complexes when the epitopes change from exposed to embedded type.

**Table 2:** 2009-H1N1 HA epitope interacting residues with DM1-TCR and HLA-B*4405 complexes.

Position	Epitope sequence	HLA/epitope (solvent-exposed residues)	TCR-HLA/epitope (interacting residues)
50	LEDKHNGKL	Lys^4^	Lys^4^
128	FERFEIFPK	Arg^3^, Glu^5^	Arg^3^
188	KEVLVLWGI	-	Lys^1^, Leu^4^, Leu^6^, Gly^8^
251	VEPGDKITF	Asp^5^, Lys^6^	Asp^5^, Lys^6^
412	KEFNHLEKR	Asn^4^, His^5^, Glu^7^	Glu^2^, Asn^4^, His^5^, Glu^7^
446	LENERTLDY	-	Leu^1^, Glu^4^, Asp^8^
463	YEKVRSQLK	Arg^5^	Arg^5^, Lys^9^
493	MESVKNGTY	Met^1^	Met^1^, Lys^5^
514	REEIDGVKL	Lys^8^	Lys^8^
131	FEIFPKTSS	Phe^1^	Phe^1^, Lys^6^
229	PEIAIRPKV	Ala^4^, Ile^5^	Ile^5^
400	IEKMNTQFT	Lys^3^, Met^4^	Glu^2^, Lys^3^, Met^4^, Asn^5^, Thr^6^
421	IENLNKKVD	-	Lys^7^, Asp^9^
475	KEIGNGCFE	-	Lys^1^, Glu^2^
482	FEFYHKCDN	-	Lys^6^
240	QEGRMNYYW	-	Asn^6^, Tyr^7^, Tyr^8^, Trp^9^
259	FEATGNLVV	Thr^4^, Gly^5^, Asn^6^	Glu^2^, Thr^4^, Gly^5^

Epitope candidates at positions 81 and 274 in 1918 and WHO vaccine sequences [Additional file 2] yielded rather low HLA-B*4405 binding scores (less than threshold 0.75) and could not be docked to DM1-TCR. Therefore it is unlikely that the two candidate epitopes were antigenic to the 1918 and WHO strains. Mutations at positions 81 and/or 274 in HA sequences of the 2009 viral strains increased the HLA-B*4405 binding scores above the threshold, but did not facilitate TCR recognition in our model.

### Recognition of computationally inferred optimal epitope KEKMNTEFW by HLA-B4405 and DM1-TCR

We computationally designed the mosaic epitope candidate KEKMNTEFW from five epitope sequences, which were in the top 5 ranks of DM1-TCR binding energy (Table 1). These epitopes are at positions 463, 482, 412, 475, and 400, whose corresponding residue positions were favourably bound by the DM1-TCR model. We selected the epitope IEKMNTQFT at position 400 as the starting point because most of its residues made direct contacts with DM1-TCR, i.e. -EKMNT---(Table 2). Also, Archbold *et al. *[12] suggested that the second positioned residue Glu (E^2^) be required for preferential binding to HLA-B44. According to our top 5 DM1-TCR docking results, 2 out of 5 epitopes (412 and 475) contain residue Lys (K) at the first position, and Lys^epitope475 ^directly interacted with the DM1-TCR. Therefore, we used Lys (K^1^) for the first position of the mosaic epitope. Similarly, we chose Glu (E) for the 7^th ^position since it was a TCR-interacting residue of the epitope 412. Finally, we used bulky side-chains of F^8 ^(Phe) and W^9 ^(Trp) serving as anchors for HLA-B*4405 binding. Thus, using Deep-View [17], we substituted residues I^1^, Q^7^, and T^9 ^with K, E, and W respectively.

Docking of the mosaic epitope KEKMNTEFW to HLA-B*4405 and DM1-TCR showed that it bound favourably to both HLA-B*4405 (binding energy -849.5 kcal/mol; rank 4) and DM1-TCR (-684.4 kcal/mol; rank 6) with Asn^5 ^and Thr^6 ^exposed to the solvent and directly interacting with DM1-TCR. Although the HA peptide appears to be a good candidate for inclusion in multivalent peptide vaccine against the H1N1 influenza A, its efficacy as a protective epitope on population level depends on the TCR repertoire which could be only tested experimentally.

### Cross-recognition of 2009-H1N1 and 2004-H5N1 HA T cell epitope candidates

A study by Kreijtz *et al. *[18] showed that T cell responses to seasonal H1N1 and H3N2 strains are largely cross-reactive to avian H5N1. According to WHO Global Influenza Program [19], H5 HA viral strain A/Vietnam/1194/2004 of the avian flu outbreak in Vietnam was one of the H5N1 prototype vaccine strains in 2005 and recommended candidate of pre-pandemic H5N1 vaccine. Therefore, we used HA protein of this strain as a model to test if our 2009-H1N1 T cell epitope candidates would be cross-reactive for 2004-H5N1.

NetCTL v1.2 was used to extract the 2004-H5N1 HLA-B*4405 restricted T cell epitopes candidates from IEDB. Of all candidates with predicted scores greater than 0.75, seven were highly similar (greater than 78% similarity) to the 2009-H1N1 T cell epitope candidates (Table 3) and likely to be recognized by DM1-TCR.

**Table 3:** Seven 2009-H1N1 T cell epitope candidates that could be cross-reactive with 2004-H5N1 T cell responses.

2009_H1N1 position	Strain	Sequence	MHC-bound predicted score	Docked to HLA-B*4405	Docked to DM1-TCR
				
				Energy	Energy
50	2009-H1N1	LED**K**HNGKL	1.0508	-661.4	**-552.1**
	2004-H5N1	LEKTHNG**KL**	1.1478	-715.0	**-682.2**
412	2009-H1N1	KEFN**H**LEKR	0.9764	-599.5	**-737.2**
	2004-H5N1	REFN**N**LEKR	0.9949	-638.9	**-640.8**
446	2009-H1N1	**L**EN**E**RTL**D**Y	1.1929	-603.1	-621.1
	2004-H5N1	M**E**N**ER**T**LDF**	1.5813	-563.5	-667.2
493	2009-H1N1	**M**ESV**K**NGTY	1.73	-543.0	-645.0
	2004-H5N1	M**ES**VR**N**G**TY**	1.6645	-595.0	-667.5
421	2009-H1N1	IENLNK**K**V**D**	0.8563	-464.6	-666.0
	2004-H5N1	IENLNK**KME**	0.7968	-466.1	-642.0
475	2009-H1N1	**KE**IGNGCFE	0.9892	-519.6	-715.4
	2004-H5N1	**K**ELGNGC**FE**	0.8258	-548.9	-733.8
482	2009-H1N1	FEFYH**K**CDN	1.1128	-846.8	-743.9
	2004-H5N1	FEFYH**K**CDN	1.1129	-846.8	-743.9

*Different residues are underlined. The residues that interacted with DM1-TCR are highlighted in bold.

Two significant changes in TCR binding energy are marked in bold.

Docking of the seven HLA-B*4405/2004-H5N1 epitope complexes to DM1-TCR revealed that the DM1-TCR predominantly interacted with the 2004-H5N1 epitopes at conserved positions 50, 446, 493, and 475. DM1-TCR binding energies of the 2004-H5N1 epitope candidates were lower and thus more favourable than these of the corresponding 2009-H1N1 epitope candidates (Table 3). Most mutations that occurred between these two influenza outbreaks did not appear to affect DM1-TCR recognition of HLA-B*4405 presented epitope candidates.

**Figure 1:**
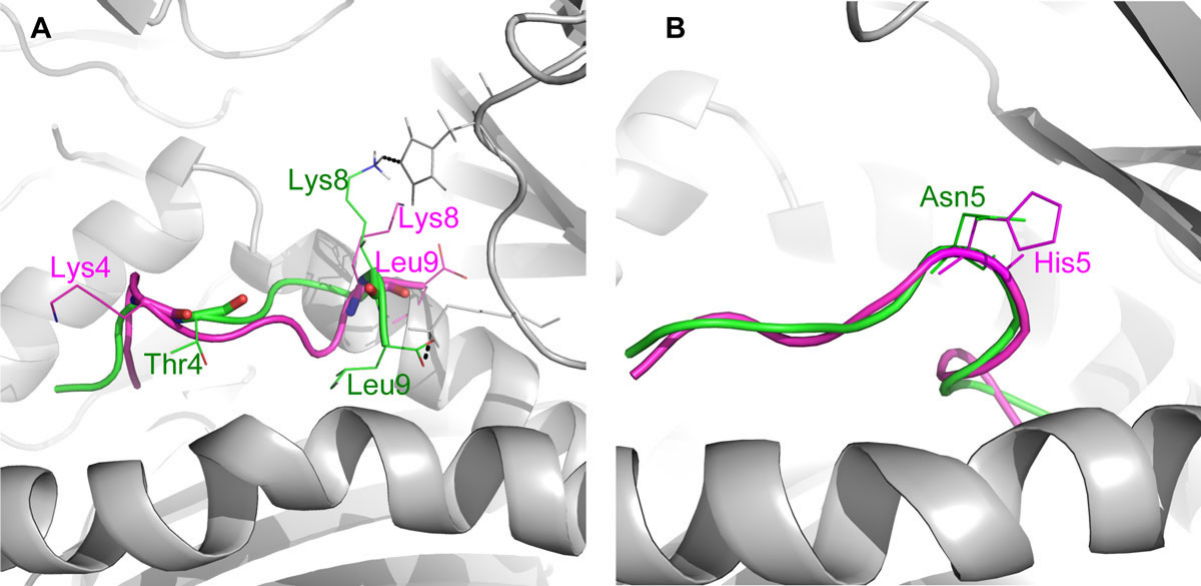
Conformational changes of DM1-TCR/HLA-B*4405/epitope complexes caused by epitope mutations. (A) Mutation K4T in the exposed region of 2004-H5N1 epitope 50 induced interaction of the C-terminal Lys^8 ^and Leu^9 ^with DM1-TCR. (B) Reduced exposed DM1-TCR (grey) contact area caused by mutation H5N of 2004-H5N1 epitope 412. Epitopes are shown in green (2004-H5N1) and magenta (2009-H1N1). Residue side chains are presented as lines.

However, we observed a significant decrease in TCR binding energy of the epitope candidate 2004-H5N1 complex at position 50 (Table 3). The mutation K4T changed the structural conformation of the epitope's exposed region. The side chain of the mutated Thr^4 ^pointed toward the HLA-α_2 _domain and is embedded in the helical binding groove of the HLA-B*4405. It might therefore induce the interactions of Lys^8 ^and Leu^9 ^at its C-terminal with the DM1-TCR (Figure 1A). This could consequently suggest a more favourable binding of DM1-TCR to the HLA-B*4405/epitope50 in 2004-H5N1 than in 2009-H1N1 viral strain.

In addition, we noticed that the binding affinity of DM1-TCR to the 2004-H5N1 epitope candidates considerably decreased when the TCR interactions occurred directly at mutated residues of the epitope candidates at position 412 (H5N) and 421 (V8M and D9E). Substitution of a positively charged His with a smaller-sized neutral Asn reduced the contact surface of the epitope candidate's exposed region (Figure 1B), resulting in a conformationally constrained contact of DM1-TCR with 2004-H5N1 candidate epitope 412 and a higher binding energy (-640.8 kcal/mol) compared to the 2009-H1N1 candidate epitope 412. In a future pandemic we expect that apart from a few mutated epitopes, heterologous immunity [20] mediated by pre-existing cross-reactive T cell responses to seasonal influenza virus will limit its severity and extent.

## Conclusions

The HLA-B*4405 and DM1-TCR docking models showed differential recognition of the 2009-H1N1 HA T cell epitope candidates, reflecting the topological constraints of the epitopes. This information was used to derive the synthetic H1N1 epitope KEKMNTEFW with optimal recognition of both HLA-B*4405 and DM1-TCR models and to identify likely cross-reactive 2004-H5N1 epitopes. While the results are novel with regard to HLA-B*4405-restricted H1N1 HA epitopes, their utility in vaccination strategies against influenza viruses is limited by the fact that the T cell responses to viruses depend on the TCR repertoire, and in particular on the nature of TCR alpha chain and their conformation as shown in a study by Zhong et al. [21]. To simulate a T cell response to H1N1 epitopes on population level as it is desirable for vaccine design, a large number of crystal structure data on TCR Vα and Vβ chains and their heterodimers would be necessary to computationally assess epitope candidates for their potential to induce a broad T cell response.

## Methods

### Obtaining HA candidate epitopes from pandemic (H1N1) 2009 sequences

Potential epitopes were predicted using T cell epitope prediction tool (NetCTL v1.2) from IEDB [22,23]. NetCTL, a neural network architecture-based tool, was used to predict T cell epitope candidates for HA proteins of current H1N1 influenza A strains according to HLA-B44 supertype. Weight parameters on C-terminal cleavage (0.15) and transporter associated with antigen processing (TAP) efficiency (0.05) were as default. There are 3 threshold scores (*0.75*, *0.9*, *1.0*) that give both high sensitivity (0.8, 0.74, 0.7) and specificity (0.97, 0.98, 0.985) accordingly. While the first 2 scores (*0.75 *and *0.9*) obtained similar results of T cell epitopes, the score of *1.0 *resulted only 7 epitopes (positions 240, 188, 128, 482, 400, 131, and 50) shown in Table 1. Our docking results of HLA-B*4405 and the T cell epitope candidates showed that epitope candidates at positions 259, 251, and 229, which were located in the top 10 HLA-binding energy rankings in Table 1, would be missed if the *1.0 *score was used.

Finally, as comparing to the score of *0.9*, we selected *0.75 *as the threshold for its better sensitivity (0.8) to predict the potential T cell epitopes. Therefore candidates with prediction scores greater than *0.75 *were chosen for further investigation. I-TASSER [24] was used to obtain homology models of the selected T cell epitopes.

### Molecular docking of predicted HLA-B*4405-binding epitopes to T cell receptor DM1-TCR

The pipeline included docking the epitopes to HLA-B*4405 [PDB:3DX8] and followed by docking of the HLA/epitope complex as a ligand to DM1-TCR [PDB:3DXA]. The binding ability of the predicted epitopes was further analyzed using ClusPro v.2 [25,26].

ClusPro v.2 is a web-based automated docking program performing a multistage protocol: rigid PIPER docking, filtering and clustering of docked conformations, and stabilizing using Monte Carlo simulations [26]. During the docking, HLA β-domain was masked but remained surface accessible since it is not involved in interaction with the TCR [13]. The results were clustered according to their binding energies. The binding energy score (hereby called "binding energy") is generated from an energy function of PIPER docking program. This function is a sum of potential terms of shape complementarity, electrostatics, desolvation contributions, and Decoys as reference states (DARS) [25]. According to Kozakov et al., the core idea of the knowledge-based potential DARS counts on observed numbers of intermolecular interactions [25]. Therefore we filtered our docking results by selecting docked complexes that belonged to the most populated clusters of interacting complexes but with lowest binding energy scores for our final results. We assumed that the model with minimum binding energy was the optimal conformation.

Then, we performed molecular dynamics (MD) simulations using AMBER 10 force field *ff99SB *[27] to improve the bound conformation of rigid docking. The TCR-HLA/epitope docked complexes underwent a 3-stage MD simulation (minimization, heating, and equilibration) using explicit solvent model under periodic boundary condition. In the minimization process, we applied a weak positional restraint using a force constant of 500 kcal/molÅ^2 ^on the whole complex during the first 1,000 steps under restrained conditions, while initially minimizing positions of solvent and sodium ions. For the subsequent 2,500 steps of minimization, we removed this restraint. The constant volume was set during both the 2 stages of the minimization process. In the heating stage of 20 ps, we restrained the complex again, but with only 10 kcal/molÅ^2 ^to avoid wild fluctuations in the structure. We allowed the system to heat up from 0 K to 300 K and applied the Langevin temperature equilibration scheme to control the temperature. Then, a short equilibration stage (1ns) without the restraints was performed in constant pressure of 1 atm and at 300 K. We used SHAKE in both heating and equilibration stages to constrain bonds that involves hydrogen. As a result, complexes obtained from the MD simulation above were considered final bound conformations of the docked TCR-HLA/epitope complexes in our study.

## List of abbreviations

Some abbreviations were used in the text: HA: (Hemagglutinin); HLA: (Human leukocyte antigen); TCR: (T cell Receptor); CTL: (Cytotoxic T Lymphocyte); MHC: (Major histocompatibility complex); TAP: (Transporter associated with antigen processing); DARS: (Decoys as the Reference States); MD: (Molecular Dynamics).

## Competing interests

The authors declare that they have no competing interests.

## Authors' contributions

CTTS carried out molecular docking experiments. CTTS, CS and CKK wrote the manuscript. All authors read and approved the final manuscript.

## Funding

This work was supported by Singapore MOE AcRF Grant No: MOE2008-T2-1-1074 and Jardine OneSolution JOS-M4060054.

## Declarations

Funding for publication of this work was supported by Singapore MOE AcRF Grant No: MOE2008-T2-1-1074 and Jardine OneSolution JOS-M4060054.

This article has been published as part of *BMC Bioinformatics *Volume 14 Supplement 2, 2013: Selected articles from the Eleventh Asia Pacific Bioinformatics Conference (APBC 2013): Bioinformatics. The full contents of the supplement are available online at http://www.biomedcentral.com/bmcbioinformatics/supplements/14/S2.

## Supplementary Material

Additional file 1 **MAFFT multiple sequence alignment of pandemic strains (2009 - the top 7 sequences) HA proteins to HA (1918 - South Carolina) and WHO vaccine HA (the last 3 sequences)**. Click here for file

Additional file 2 **Predicted HA T cell epitopes of H1N1 2009 strains compared to 1918 and WHO-1999-2006-2007 strains**. Click here for file

Additional file 3 **Physicochemical properties of NetCTL-predicted HLA-B44 restricted T cell epitope candidates (A) and non-epitopes (B)**. Click here for file

Additional file 4 Residues of domain α1, α2 of HLA-B*4405 interacting with DM1-TCR. Click here for file

## References

[B1] Smith GJD Vijaykrishna D Bahl J Lycett SJ Worobey M Pybus OG Ma SK Cheung CL Raghwani J Bhatt S Origins and evolutionary genomics of the 2009 swine-origin H1N1 influenza A epidemic Nature 2009 459 1122 1125 10.1038/nature08182 19516283

[B2] Gatherer D The 2009 H1N1 influenza outbreak in its historical context J Clin Virol 2009 45 3 174 178 10.1016/j.jcv.2009.06.004 19540156

[B3] Gras S Kedzierski L Valkenburg S Laurie K Liu Y Denholm J Richards M Rimmelzwaan G Kelso A Doherty P Cross-reactive CD8+ T-cell immunity between the pandemic H1N1-2009 and H1N1-1918 influenza A viruses Proc Natl Acad Sci USA 2010 107 28 12599 12604 10.1073/pnas.1007270107 20616031 PMC2906563

[B4] Xu R Ekiert DC Krause JC Hai R Crowe JE Jr Wilson IA Structural Basis of Preexisting Immunity to the 2009 H1N1 Pandemic Influenza Virus Science 2010 328 357 360 10.1126/science.1186430 20339031 PMC2897825

[B5] Boon ACM Mutsert Gd Baarle Dv Smith DJ Lapedes AS Fouchier RAM Sintnicolaas K Osterhaus ADME Rimmelzwaan GF Recognition of homo- and heterosubtypic variants of influenza A viruses by human CD8+ T lymphocytes The Journal of Immunology 2004 172 2453 2460 14764717 10.4049/jimmunol.172.4.2453

[B6] Subbramanian R Basha S Shata M Brady R Bernstein D Pandemic and seasonal H1N1 influenza hemagglutinin-specific T-cell responses elicited by seasonal influenza vaccination Vaccine 2010 28 52 8258 8267 10.1016/j.vaccine.2010.10.077 21050903

[B7] Assarsson E Bui H-H Sidney J Zhang Q Glenn J Oseroff C Mbawuike IN Alexander J Newman MJ Grey H Immunomic analysis of the repertoire of T-cell specificities for Influenza A Virus in Humans Journal of Virology 2008 82 24 12241 12251 10.1128/JVI.01563-08 18842709 PMC2593359

[B8] Groot ASD Ardito M McClaine EM Moise L Martin WD Immunoinformatic comparison of T-cell epitopes contained in novel swine-origin influenza A (H1N1) virus with epitopes in 2008-2009 conventional influenza vaccine Vaccine 2009 27 5740 5747 10.1016/j.vaccine.2009.07.040 19660593

[B9] Gupta SK Srivastava M Akhoon BA Smita S Schmitz U Wolkenhauer O Vera J Gupta SK Identification of immunogenic consensus T-cell epitopes in globally distributed influenza A H1N1 neuraminidase Infect Genet Evol 2010 11 2 308 319 21094280 10.1016/j.meegid.2010.10.013

[B10] Wang JH Reinherz EL Structural basis of T cell recognition of peptides bound to MHC molecules Mol Immunol 2002 38 1039 1049 10.1016/S0161-5890(02)00033-0 11955596

[B11] Chang H-C Smolyar A Spoerl R Witte T Yao Y Goyarts EC Nathenson SG Reinherz EL Topology of T cell Receptor-peptide/Class I MHC interaction defined by charge reversal complementation and functional analysis J Mol Biol 1997 271 278 293 10.1006/jmbi.1997.1169 9268659

[B12] Archbold JK Macdonald WA Gras S Ely LK Miles JJ Bell MJ Brennan RM Beddoe T Wilce MCJ Clements CS Natural micropolymorphism in human leukocyte antigens provides a basis for genetic control of antigen recognition J Exp Med 2009 206 1 209 219 10.1084/jem.20082136 19139173 PMC2626662

[B13] Reinherz EL Tan K Tang L Kern P Liu J-h Xiong Y Hussey RE Smolyar A Hare B Zhang R The Crystal Structure of a T Cell Receptor in Complex with Peptide and MHC Class II Science 1999 286 1913 1921 10.1126/science.286.5446.1913 10583947

[B14] DiBrino M Parker KC Margulies DH Shiloach J Turner RV Biddison WE Coligan JE Identification of the peptide binding motif for HLA-B44, one of the most common HLA-B alleles in the Caucasian Population Biochemistry 1995 34 10130 10138 10.1021/bi00032a005 7543776

[B15] Macdonald WA Purcell AW Mifsud NA Ely LK Williams DS Chang L Gorman JJ Clements CS Kjer-Nielsen L Koelle DM A Naturally Selected Dimorphism within the HLA-B44 Supertype Alters Class I Structure, Peptide Repertoire, and T Cell Recognition The Journal of Experimental Medicine 2003 198 5 679 691 10.1084/jem.20030066 12939341 PMC2194191

[B16] Wahl A McCoy W Schafer F Bardet W Buchli R Fremont DH Hildebrand WH T-Cell Tolerance for Variability in an HLA Class I-Presented Influenza A Virus Epitope Journal of Virology 2009 83 18 9206 9214 10.1128/JVI.00932-09 19553306 PMC2738244

[B17] Guex N Peitsch MC Swiss-Model and the Swiss-Pdb Viewer: An environment for comparative protein modeling Electrophoresis 1997 18 2714 2723 10.1002/elps.1150181505 9504803

[B18] Kreijtz JHCM Mutsert Gd Baalen CAv Fouchier RAM Osterhaus ADME Rimmelzwaan GF Cross-Recognition of Avian H5N1 Influenza Virus by Human Cytotoxic T-Lymphocyte Populations Directed to Human Influenza A Virus Journal of Virology 2008 82 11 5161 5166 10.1128/JVI.02694-07 18353950 PMC2395172

[B19] Availability of new H5N1 prototype strain for influenza pandemic vaccine development http://www.who.int/influenza/vaccines/virus/2strains2006/en/

[B20] Selin LK Welsh RM Plasticity of T Cell Memory Responses to Viruses Immunity 2004 20 5 16 10.1016/S1074-7613(03)00356-X 14738760 PMC7130098

[B21] Zhong W Dixit SB Mallis RJ Arthanari H Lugovskoy AA Beveridge DL Wagner G Reinherz EL CTL Recognition of a Protective Immunodominant Influenza A Virus Nucleoprotein Epitope Utilizes a Highly Restricted Vβ but Diverse Vα Repertoire: Functional and Structural Implications J Mol Biol 2007 372 535 548 10.1016/j.jmb.2007.06.057 17658550

[B22] Larsen M Lundergard C Lamberth K Buus S Brunak S Lund O Nielsen M An integrative approach to CTL epitope prediction: A combined algorithm integrating MHC class I binding, TAP transport efficiency, and proteasomal cleavage predictions European Journal of Immunology 2005 35 8 2295 2303 10.1002/eji.200425811 15997466

[B23] Larsen M Lundegaard C Lamberth K Buus S Lund O Nielsen M Large-scale validation of methods for cytotoxic T-lymphocyte epitope prediction BMC Bioinformatics 2007 8 1 424 10.1186/1471-2105-8-424 17973982 PMC2194739

[B24] Roy A Kucukural A Zhang Y I-TASSER: a unified platform for automated protein structure and function prediction Nat Protoc 2010 5 4 725 738 10.1038/nprot.2010.5 20360767 PMC2849174

[B25] Kozakov D Brenke R Comeau SR Vajda S PIPER: An FFT-based protein docking program with pairwise potentials Proteins 2006 65 392 406 10.1002/prot.21117 16933295

[B26] Kozakov D Hall DR Beglov D Branke R Comeau SR Shen Y Li K Zheng J Vakili P Paschalidis IC Achieving reliability and high accuracy in automated protein docking: ClusPro, PIPER, SDU, and stability analysis in CAPRI rounds 13-19 Proteins 2010 78 3124 3130 10.1002/prot.22835 20818657 PMC3027207

[B27] Case DA Cheatham TE III Darden T Gohlke H Luo R Merz KM Jr Onufriev A Simmerling S Wang B Woods R The Amber biomolecular simulation programs J Computat Chem 2005 26 1668 1688 10.1002/jcc.20290 PMC1989667 16200636

