# Supplementary material for: Molecular docking analysis of 2009-H1N1 and 2004-H5N1 influenza virus HLA-B*4405-restricted HA epitope candidates: implications for TCR cross-recognition and vaccine development
Source: BMC Bioinformatics. 2013 Jan 21;14(Suppl 2):S21. doi: 10.1186/1471-2105-14-S2-S21 (PMC3549837; doi:10.1186/1471-2105-14-S2-S21)
Supplement: Additional file 1 — MAFFT multiple sequence alignment of pandemic strains (2009 - the top 7 sequences) HA proteins to HA (1918 - South Carolina) and WHO vaccine HA (the last 3 sequences). [file 1471-2105-14-S2-S21-S1.pdf]

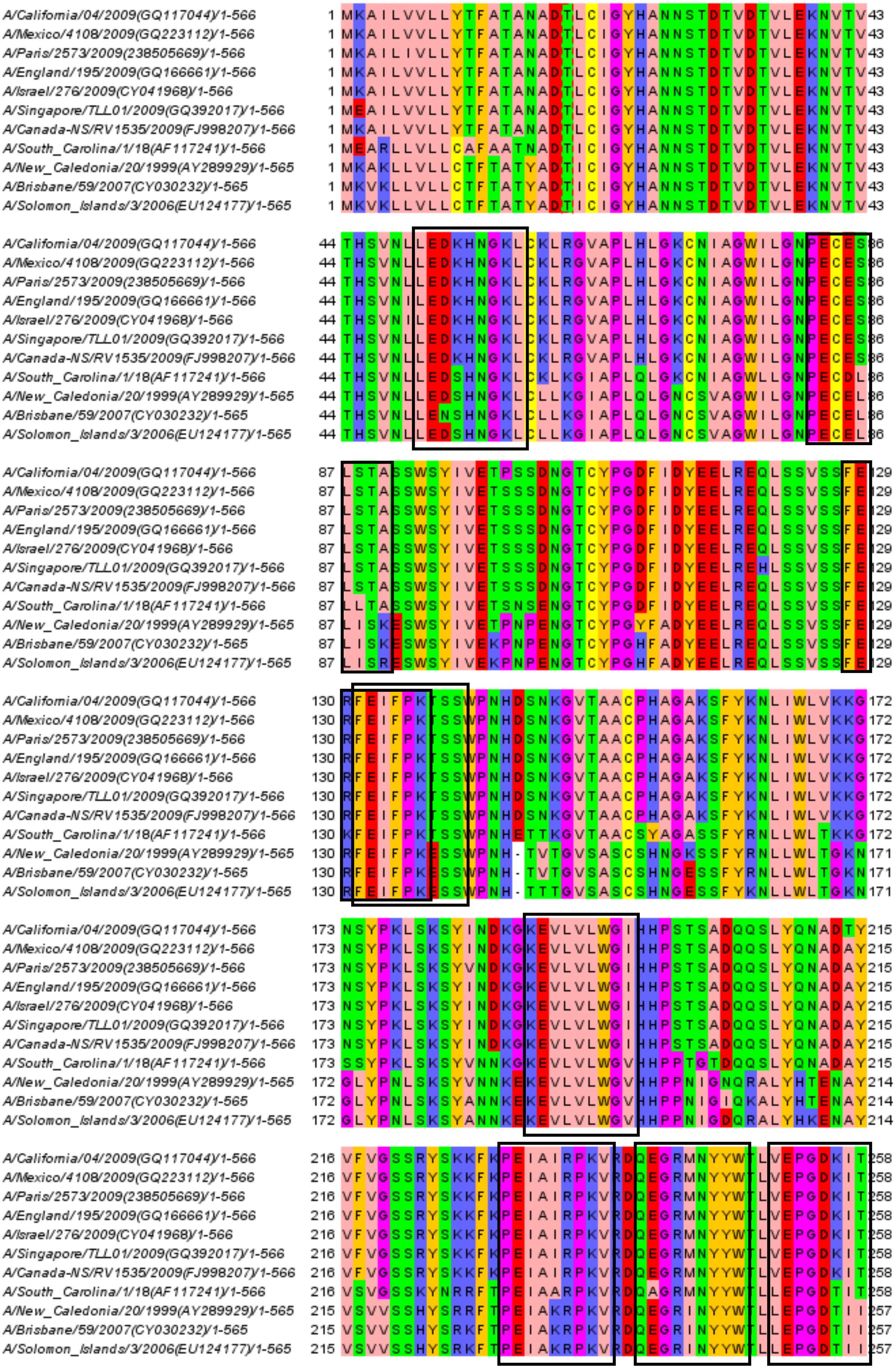
A/California/04/2009(GQ117044)/1-566
A/Mexico/4108/2009(GQ223112)/1-566
A/Paris/2573/2009(238505669)/1-566
A/England/195/2009(GQ166661)/1-566
A/Israel/276/2009(CY041968)/1-566
A/Singapore/TLL01/2009(GQ392017)/1-566
A/Canada-NS/RV1535/2009(FJ998207)/1-566
A/South_Carolina/1/18(AF117241)/1-566
A/New_Caledonia/20/1999(AY289929)/1-565
A/Brisbane/59/2007(CY030232)/1-565
A/Solomon_Islands/3/2006(EU124177)/1-565

Sequence	Start	Alignment	End
A/California/04/2009(GQ117044)/1-566	259	FEATGNLVVPRYAFAMERNAGSGIIISDTPVHDCNTTCQTPKG	301
A/Mexico/4108/2009(GQ223112)/1-566	259	FEATGNLVVPRYAFAMERNAGSGIIISDTPVHDCNTTCQTPKG	301
A/Paris/2573/2009(238505669)/1-566	259	FEATGNLVVPRYAFAMERNAGSGIIISDTPVHDCNTTCQTPKG	301
A/England/195/2009(GQ166661)/1-566	259	FEATGNLVVPRYAFAMERNAGSGIIISDTPVHDCNTTCQTPKG	301
A/Israel/276/2009(CY041968)/1-566	259	FEATGNLVVPRYAFAMERNAGSGIIISDTPVHECNTTCQTPKG	301
A/Singapore/TLL01/2009(GQ392017)/1-566	259	FEATGNLVVPRYAFAMERNAGSGIIISDTPVHDCNTTCQTPKG	301
A/Canada-NS/RV1535/2009(FJ998207)/1-566	259	FEATGNLVVPRYAFAMERNAGSGIIISDTPVHDCNTTCQTPKG	301
A/South_Carolina/1/18(AF117241)/1-566	259	FEATGNLIAPWYAFALNRGSGSGIITSDAPVHDCNTKCQTPHG	301
A/New_Caledonia/20/1999(AY289929)/1-565	258	FEANGNLIAPWYAFALSRGFGSGIITSNAPMDECDAKCQTPQG	300
A/Brisbane/59/2007(CY030232)/1-565	258	FEANGNLIAPRYAFALSRGFGSGIINSNAPMDKCDAKCQTPQG	300
A/Solomon_Islands/3/2006(EU124177)/1-565	258	FEANGNLIAPRYAFALSRGFGSGIINSNAPMDECDAKCQTPQG	300

Sequence	Start	Alignment	End
A/California/04/2009(GQ117044)/1-566	302	AINTSLPFQNIHPITIGKCPKYVKSTKLRLATGLRNIPSIQSR	344
A/Mexico/4108/2009(GQ223112)/1-566	302	AINTSLPFQNIHPITIGKCPKYVKSTKLRLATGLRNVPSIQSR	344
A/Paris/2573/2009(238505669)/1-566	302	AINTSLPFQNIHPITIGKCPKYVKSTKLRLATGLRNVPSIQSR	344
A/England/195/2009(GQ166661)/1-566	302	AINTSLPFQNIHPITIGKCPKYVKSTKLRLATGLRNVPSIQSR	344
A/Israel/276/2009(CY041968)/1-566	302	AINTSLPFQNIHPITIGKCPKYVKSTKLRLATGLRNVPSIQSR	344
A/Singapore/TLL01/2009(GQ392017)/1-566	302	AINTSLPFQNIHPITIGKCPKYVKSTKLRLATGLRNVPSIQSR	344
A/Canada-NS/RV1535/2009(FJ998207)/1-566	302	AINTSLPFQNIHPITIGKCPKYVKSTKLRLATGLRNVPSIQSR	344
A/South_Carolina/1/18(AF117241)/1-566	302	AINSSLPFQNIHPVTIGECPKYVRSTKLRMATGLRNIPSIQSR	344
A/New_Caledonia/20/1999(AY289929)/1-565	301	AINSSLPFQNVHPVTIGECPKYVRSAKLRMVTGLRNIPSIQSR	343
A/Brisbane/59/2007(CY030232)/1-565	301	AINSSLPFQNVHPVTIGECPKYVRSAKLRMVTGLRNIPSIQSR	343
A/Solomon_Islands/3/2006(EU124177)/1-565	301	AINSSLPFQNVHPVTIGECPKYVRSAKLRMVTGLRNIPSIQSR	343

Sequence	Start	Alignment	End
A/California/04/2009(GQ117044)/1-566	345	GLFGAIAGFIEGGWTGMVDGWYGYHHQNEQGSGYAADLKSTQN	387
A/Mexico/4108/2009(GQ223112)/1-566	345	GLFGAIAGFIEGGWTGMVDGWYGYHHQNEQGSGYAADLKSTQN	387
A/Paris/2573/2009(238505669)/1-566	345	GLFGAIAGFIEGGWTGMVDGWYGYHHQNEQGSGYAADLKSTQN	387
A/England/195/2009(GQ166661)/1-566	345	GLFGAIAGFIEGGWTGMVDGWYGYHHQNEQGSGYAADLKSTQN	387
A/Israel/276/2009(CY041968)/1-566	345	GLFGAIAGFIEGGWTGMVDGWYGYHHQNEQGSGYAADLKSTQN	387
A/Singapore/TLL01/2009(GQ392017)/1-566	345	GLFGAIAGFIEGGWTGMVDGWYGYHHQNEQGSGYAADLKSTQN	387
A/Canada-NS/RV1535/2009(FJ998207)/1-566	345	GLFGAIAGFIEGGWTGMVDGWYGYHHQNEQGSGYAADLKSTQN	387
A/South_Carolina/1/18(AF117241)/1-566	345	GLFGAIAGFIEGGWTGMIDGWYGYHHQNEQGSGYAADQKSTQN	387
A/New_Caledonia/20/1999(AY289929)/1-565	344	GLFGAIAGFIEGGWTGMVDGWYGYHHQNEQGSGYAADQKSTQN	386
A/Brisbane/59/2007(CY030232)/1-565	344	GLFGAIAGFIEGGWTGMVDGWYGYHHQNEQGSGYAADQKSTQN	386
A/Solomon_Islands/3/2006(EU124177)/1-565	344	GLFGAIAGFIEGGWTGMVDGWYGYHHQNEQGSGYAADQKSTQN	386

Sequence	Start	Alignment	End
A/California/04/2009(GQ117044)/1-566	388	AIDEITNKVNSVIEKMNTQFTAVGKEFNHLEKRIENLNKKVDD	430
A/Mexico/4108/2009(GQ223112)/1-566	388	AIDEITNKVNSVIEKMNTQFTAVGKEFNHLEKRIENLNKKVDD	430
A/Paris/2573/2009(238505669)/1-566	388	AIDEITNKVNSVIEKMNTQFTAVGKEFNHLEKRIENLNKKVDD	430
A/England/195/2009(GQ166661)/1-566	388	AIDEITNKVNSVIEKMNTQFTAVGKEFNHLEKRIENLNKKVDD	430
A/Israel/276/2009(CY041968)/1-566	388	AIDEITNKVNSVIEKMNTQFTAVGKEFNHLEKRIENLNKKVDD	430
A/Singapore/TLL01/2009(GQ392017)/1-566	388	AIDEITNKVNSVIEKMNTQFTAVGKEFNHLEKRIENLNKKVDD	430
A/Canada-NS/RV1535/2009(FJ998207)/1-566	388	AIDEITNKVNSVVEKMNTQFTAVGKEFNHLEKRIENLNKKVDD	430
A/South_Carolina/1/18(AF117241)/1-566	388	AIDGITNKVNSVIEKMNTQFTAVGKEFNNLERRIENLNKKVDD	430
A/New_Caledonia/20/1999(AY289929)/1-565	387	AINGITNKVNSVIEKMNTQFTAVGKEFNKLERRMENLNKKVDD	429
A/Brisbane/59/2007(CY030232)/1-565	387	AINGITNKVNSVIEKMNTQFTAVGKEFNKLERRMENLNKKVDD	429
A/Solomon_Islands/3/2006(EU124177)/1-565	387	AINGITNKVNSVIEKMNTQFTAVGKEFNKLERRMENLNKKVDD	429

Sequence	Start	Alignment	End
A/California/04/2009(GQ117044)/1-566	431	GFLDIWTYNAELLVLLENERTLDYHDSNVKNLYEKVRSQLKNN	473
A/Mexico/4108/2009(GQ223112)/1-566	431	GFLDIWTYNAELLILLENERTLDYHDSNVKNLYEKVRSQLKNN	473
A/Paris/2573/2009(238505669)/1-566	431	GFLDIWTYNAELLVLLENERTLDYHDSNVKNLYEKVRSQLKNN	473
A/England/195/2009(GQ166661)/1-566	431	GFLDIWTYNAELLVLLENERTLDYHDSNVKNLYEKVRSQLKNN	473
A/Israel/276/2009(CY041968)/1-566	431	GFLDIWTYNAELLVLLENERTLDYHDSNVKNLYEKVRSQLKNN	473
A/Singapore/TLL01/2009(GQ392017)/1-566	431	GFLDIWTYNAELLVLLENERTLDYHDSNVKNLYEKVRSQLKNN	473
A/Canada-NS/RV1535/2009(FJ998207)/1-566	431	GFLDIWTYNAELLVLLENERTLDYHDSNVKNLYEKVRSQLKNN	473
A/South_Carolina/1/18(AF117241)/1-566	431	GFLDIWTYNAELLVLLENERTLDFHDSNVRNLYEKVKSQLKNN	473
A/New_Caledonia/20/1999(AY289929)/1-565	430	GFLDIWTYNAELLVLLENERTLDFHDSNVKNLYEKVKSQLKNN	472
A/Brisbane/59/2007(CY030232)/1-565	430	GFIDIWTYNAELLVLLENERTLDFHDSNVKNLYEKVKSQLKNN	472
A/Solomon_Islands/3/2006(EU124177)/1-565	430	GFIDIWTYNAELLVLLENERTLDFHDSNVKNLYEKVKSQLKNN	472

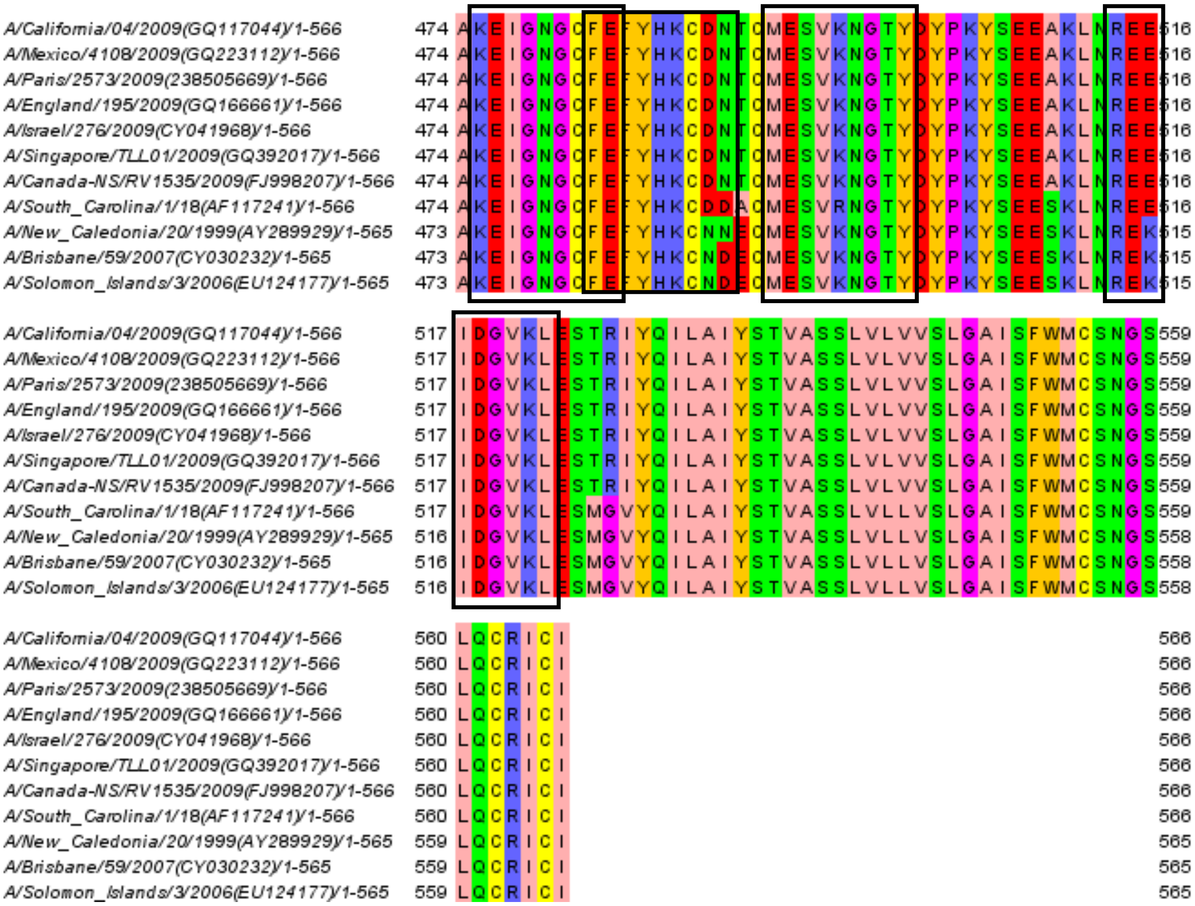

Additional file 1. MAFFT multiple sequence alignment of pandemic strains (2009 – the top 7 sequences) HA proteins to HA (1918 – South Carolina) and WHO vaccine HA (the last 3 sequences).
