# Supplementary material for: Molecular docking analysis of 2009-H1N1 and 2004-H5N1 influenza virus HLA-B*4405-restricted HA epitope candidates: implications for TCR cross-recognition and vaccine development
Source: BMC Bioinformatics. 2013 Jan 21;14(Suppl 2):S21. doi: 10.1186/1471-2105-14-S2-S21 (PMC3549837; doi:10.1186/1471-2105-14-S2-S21)
Supplement: Additional file 3 — Physicochemical properties of NetCTL-predicted HLA-B44 restricted T cell epitope candidates (A) and non-epitopes (B). [file 1471-2105-14-S2-S21-S3.pdf]

A B

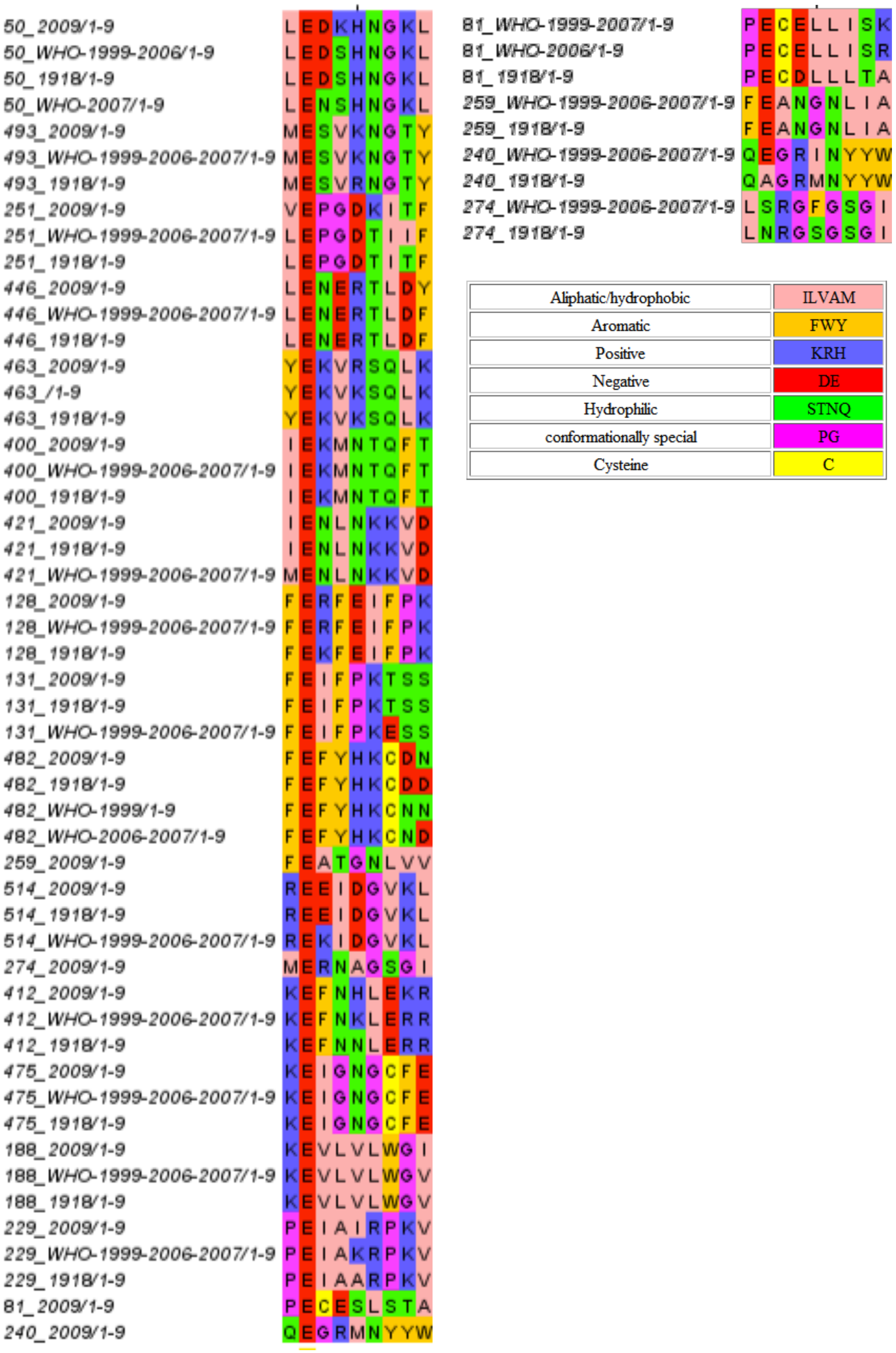

Aliphatic/hydrophobic	ILVAM
Aromatic	FWY
Positive	KRH
Negative	DE
Hydrophilic	STNQ
conformationally special	PG
Cysteine	C

Additional file 2. Physicochemical properties of NetCTL-predicted HLA-B44 restricted T cell epitope candidates (A) and non-epitopes (B).
