# Supplementary material for: Molecular docking analysis of 2009-H1N1 and 2004-H5N1 influenza virus HLA-B*4405-restricted HA epitope candidates: implications for TCR cross-recognition and vaccine development
Source: BMC Bioinformatics. 2013 Jan 21;14(Suppl 2):S21. doi: 10.1186/1471-2105-14-S2-S21 (PMC3549837; doi:10.1186/1471-2105-14-S2-S21)
Supplement: Additional file 4 — Residues of domain α1, α2 of HLA-B*4405 interacting with DM1-TCR. [file 1471-2105-14-S2-S21-S4.pdf]

Additional file 4. Residues of domain α1, α2 of HLA-B*4405 interacting with DM1-TCR

exposed/embedded	HLA-α_1	HLA-α_2	TCR V_α	TCR V_β	H-bond Length (Å)	Dihedral (°)
259	Asn^{70}		Glu^{70}		2.1	32.3
	Glu^{76}		Arg^{80}		2.2	-54.1
240	Asn^{70}			Pro^{25}	2.5	-119.7
	Glu^{76}			Gln^{6}	2.5	-133.7
400		Gln^{155}	Gln^{54}		2.2	-16.3
482		Gln^{155}		Ser^{27}	2.5	84.1
